# Molecular Phenotypes in Triple Negative Breast Cancer from African American Patients Suggest Targets for Therapy

**DOI:** 10.1371/journal.pone.0071915

**Published:** 2013-11-18

**Authors:** Robert Lindner, Catherine Sullivan, Onyinye Offor, Kimberly Lezon-Geyda, Kyle Halligan, Neal Fischbach, Mansi Shah, Veerle Bossuyt, Vincent Schulz, David P. Tuck, Lyndsay N. Harris

**Affiliations:** 1 Department of Pathology, Yale University School of Medicine, New Haven, Connecticut, United States of America; 2 Medical Oncology, Yale Cancer Center, New Haven, Connecticut, United States of America; 3 Department of Oncology, Bridgeport Hospital, Bridgeport, Connecticut, United States of America; 4 Department of Pediatrics, Yale University School of Medicine, New Haven, Connecticut, United States of America; 5 Institute of Pharmacy and Molecular Biotechnology, University of Heidelberg, Heidelberg, Germany; 6 University Hospitals, Case Western Reserve University, Cleveland, Ohio, United States of America; Dartmouth, United States of America

## Abstract

Triple negative breast cancer (TNBC) is characterized by high proliferation, poor differentiation and a poor prognosis due to high rates of recurrence. Despite lower overall incidence African American (AA) patients suffer from higher breast cancer mortality in part due to the higher proportion of TNBC cases among AA patients compared to European Americans (EA). It was recently shown that the clinical heterogeneity of TNBC is reflected by distinct transcriptional programs with distinct drug response profiles in preclinical models. In this study, gene expression profiling and immunohistochemistry were used to elucidate potential differences between TNBC tumors of EA and AA patients on a molecular level.

In a retrospective cohort of 136 TNBC patients, a major transcriptional signature of proliferation was found to be significantly upregulated in samples of AA ethnicity. Furthermore, transcriptional profiles of AA tumors showed differential activation of insulin-like growth factor 1 (IGF1) and a signature of *BRCA1* deficiency in this cohort. Using signatures derived from the meta-analysis of TNBC gene expression carried out by Lehmann *et al.*, tumors from AA patients were more likely of basal-like subtypes whereas transcriptional features of many EA samples corresponded to mesenchymal-like or luminal androgen receptor driven subtypes. These results were validated in The Cancer Genome Atlas mRNA and protein expression data, again showing enrichment of a basal-like phenotype in AA tumors and mesenchymal subtypes in EA tumors. In addition, increased expression of VEGF-activated genes together with elevated microvessel area determined by the AQUA method suggest that AA patients exhibit higher tumor vascularization.

This study confirms the existence of distinct transcriptional programs in triple negative breast cancer in two separate cohorts and that these programs differ by racial group. Differences in TNBC subtypes and levels of tumor angiogenesis in AA versus EA patients suggest that targeted therapy choices should be considered in the context of race.

## Introduction

The triple negative breast cancer (TNBC) subtype is defined by the absence of estrogen, progesterone and HER2 receptor expression. TNBC accounts for 10–20% of all breast cancer cases, with an uneven distribution of prevalence among ethnicities. In African American (AA) women, TNBC is nearly twice as prevalent as in European Americans (EA), with reports ranging up to 40% of all breast cancer cases in pre-menopausal AA patients being triple negative [1].

Consistent with this observation, epidemiologic studies show substantial differences in breast cancer mortality between racial groups. In particular, pre-menopausal American women of African ancestry have a higher breast cancer-related mortality (5-year survival of 79% compared to 91% in European American patients), putting them at a higher risk despite lower breast cancer incidence [2]. This effect may be due to the increased frequency of triple negative breast cancer observed in AA patients who typically present at higher disease stage and are more likely to have lymph node metastases at similar tumor size [3]. Previous attempts to characterize differences in tumor biology between AA and EA patients have identified increased proliferation, high grade and markers of angiogenesis in AA tumors [4]. Studies on smaller cohorts suggested an increased angiogenic profile and a higher incidence of lymph node metastases in AA patients [3], [4]. However, appropriate stratification of breast cancer subtypes has not been performed. Loo and colleagues reported different patterns of genomic copy number variations (CNV) consistently associated with ethnicity in triple negative breast cancer samples [5]. This finding, together with the clinical and molecular heterogeneity of TNBC tumors lend support to the hypothesis that differences in subtype prevalence are reflected by differences in tumor biology.

TNBC presents with high grade tumors and high rates of distant recurrence, and therefore is associated with a poor prognosis. Conversely, compared to other, less aggressive breast cancer subtypes, patients with TNBC tumors achieve higher rates of pathological complete response (pCR) from neoadjuvant chemotherapy and such patients have an overall survival similar to those who reach pCR with other breast cancer subtypes. However, TNBC patients with residual disease after treatment have a significantly worse prognosis compared to non-responding tumors from other subtypes [6], [7]. This suggests that the majority of TNBC are resistant to conventional chemotherapy and that targeted therapy approaches are critical to improve outcome in this group of patients.

Recent studies have shown that the clinical heterogeneity of TNBC is reflected by transcriptional programs [8], [9]. To date, the largest published study assessing TNBC subtypes was carried out by Lehmann *et al.*, who pooled gene expression data from 21 data sets and identified six clusters, two with basal-like differentiation, two mesenchymal-like, one immune-activated subtype and one with androgen-receptor driven luminal like gene expression [9]. The phenotypic similarity of TNBC with *BRCA1* mutation associated breast cancer [10] suggests sensitivity to agents inhibiting DNA repair such as the putative PARP (poly ADP-ribose polymerase) inhibitors olaparib and iniparib. Robust response has only been shown in *BRCA1* mutation carriers [11]. Lehmann and colleagues showed in preclinical models that cisplatin sensitivity is greater in TNBC subtypes with basal-like differentiation [9]. Aberrant signaling of one or many cell surface receptor tyrosine kinases results in production of phosphatidylinositol-3-kinase (PI3K) which produces phosphatidylinositol-3,4,5-triphosphate (PIP3), a mitogenic second messenger which acts through activation of protein kinases in the AKT pathway. The rationale for targeting the AKT pathway is the frequent dysregulation not only at the receptor level but also loss of pathway inhibitors such as PTEN and hyperactivation of enhancers such as PI3K [12]. Preclinical models suggest that tumors of a mesenchymal-like subtype of TNBC may be sensitive to inhibition of this pathway [9]. Increased levels of angiogenesis and metastasis make promoters of angiogenesis such as the vascular endothelial growth factor receptor (VEGFR) promising targets currently under investigation [13], [14]. Cell lines corresponding to the rare and very distinct luminal like androgen receptor (AR) positive subtype of TNBC were sensitive to inhibition of AR, a new strategy currently undergoing clinical trials (NCT00468715).

Gene expression profiling and immunohistochemistry using AQUA technology were used to evaluate a marker of angiogenesis in a cohort of 136 patients with TNBC. We found increased microvessel area in AA compared with EA TNBC tumors, providing an additional therapeutic strategy with angiogenesis inhibiting agents in these patients. In addition, AA tumors demonstrated a basal 1 profile and decreased BRCA1 activity suggesting that platinum drugs and PARP inhibitors might be useful therapies. Unsupervised analysis revealed modules of insulin-like growth factor 1 (IGF1) signaling and high proliferation in AA relative to EA tumors. Our findings demonstrate differences between AA and EA patients that are reflected by tumor biology and suggest potential targets for treatment. These findings should be confirmed in larger studies of therapeutic agents with IGF1-receptor, PARP- and angiogenesis inhibitors as well as platinum-based drugs.

## Results

### Cohort description

Clinical data was evaluated for 136 patients with ER/PR/HER2 negative breast cancer. Median age at diagnosis was 51 years. Most patients presented at stage I or II (81%) at which AA patients were more likely to have lymph node metastases (53% for AA vs. 33% for EA) and thus to present at stage II. Survival information was available for 115 patients with a median follow-up of 5.3 years (range: 11 months to 19 years). Stage and nodal status were significant predictors of survival (p = 0.0006 and 0.0004, respectively, univariate log rank test). Lymph node status was significantly associated with young age at diagnosis across ethnicities (p = 0.01, two-tailed t-test). Clinical characteristics of the cohort are summarized in Table 1.

**Table 1:** Clinical characteristics of the Yale TNBC cohort.

136 cases	N (%)
**Survival**	
<5 year	36 (42%)
>5 year	49 (58%)
Censored/Unknown	51
**Age**	
<51	58 (44%)
> = 51	73 (56%)
Unknown	5
**Lymph Nodes**	
negative	40 (56%)
positive	50 (44%)
Unknown	46
**Stage**	
I	31 (28%)
II	58 (53%)
III	14 (13%)
IV	6 (6%)
Unknown	27
**Ethnicity**	
European American	69 (54%)
African American	50 (39%)
Hispanic	9 (7%)
Unknown	8

Percentages refer to the total number of cases with available data.

### Unsupervised analysis identifies major transcriptional signatures in TNBC tumors

Principal component analysis (PCA) was used to examine modules of shared variance in the mRNA expression data set. The biological significance of genes associated with principal components (PC) was assessed using enrichment tests in various data bases. This approach can separate noise introduced by technical artifact from variation caused by differential activation of biological processes likely responsible for the clinical heterogeneity of TNBC.

The first three components, which accounted for 25% of the total variance observed in the data set, were not associated with significant biological information that could be used to separate the cohort into distinct groups. PC 4 was negatively associated with genes involved in estrogen receptor (ER) downstream signaling and stromal tissue markers, including extracellular matrix remodeling factors and cell adhesion molecules (Table 2). Expression modules of hypoxia, angiogenesis and cell cycle deregulation were up-regulated with PC 4 (Table 3). PC 5 and 6 carried a prominent signature of immune response (Table S1). Analysis of further components did not add any new modules producing significant enrichments in gene set databases.

**Table 2:** Gene sets negatively associated with principal component 4 by enrichment analysis.

Principal Component 4 - Bottom			
Literature-Based			
	p	# genes	significant
IGF1 Ligand Yale [18]	4.52E-25	245	51
Mammary Stem Cells [16]	7.48E-07	356	34
Stromal – DTF [38]	0.00051	268	23

Gene Ontology			
cell adhesion	0.0005	7	4
extracellular matrix organization	0.0003	185	15

MSigDB			
SMID_BREAST_CANCER_BASAL_DN	2.52E-35	555	78
SMID_BREAST_CANCER_LUMINAL_A_UP	1.13E-23	70	27
SMID_BREAST_CANCER_LUMINAL_B_UP	1.49E-12	182	24
SMID_BREAST_CANCER_NORMAL_LIKE_UP	3.95E-12	378	35
DOANE_BREAST_CANCER_ESR1_UP	4.54E-11	90	19
CHARAFE_BREAST_CANCER_LUMINAL_VS_BASAL_UP	0.00545	280	18
BECKER_TAMOXIFEN_RESISTANCE_UP	0.00843	34	6

Enrichment of gene sets from the Broad Molecular Signature Database (MSigDB) [34], Gene Ontology [33] and selected publications was assessed using Fisher's Exact test for 250 probes with the lowest projection scores on principal component 4. P-values were FDR-adjusted for multiple testing.

**Table 3:** Gene sets positively associated with principal component 4 by enrichment analysis.

Principal Component 4 - Top			
Literature-Based			
	p	# genes	significant
Doxorubicin Resistance [39]	0.00953	47	7
Genomic Grade Index [17]	9.49E-10	94	20
Luminal Progenitor Cells [16]	0.000175	133	16

Gene Ontology			
M phase	4.8E-18	554	45
nuclear division	6.2E-13	389	32
mitosis	6.2E-13	389	32

MSigDB			
SOTIRIOU_BREAST_CANCER_GRADE_1_VS_3	1.68E-15	126	28
CROONQUIST_IL6_DEPRIVATION_DN	2.71E-12	69	19
SMID_BREAST_CANCER_BASAL_UP	3.42E-11	530	48
CHANG_CYCLING_GENES	1.35E-10	47	15
BENPORATH_PROLIFERATION	2.17E-08	126	20
KANG_DOXORUBICIN_RESISTANCE_UP	6.14E-08	50	13
WINNEPENNINCKX_MELANOMA_METASTASIS	8.84E-06	135	17

Enrichment of gene sets from the Broad Molecular Signature Database (MSigDB) [34], Gene Ontology [33] and selected publications was assessed using Fisher's Exact test for 250 probes with the highest projection scores on principal component 4. P-values were FDR-adjusted for multiple testing.

**Figure 1:**
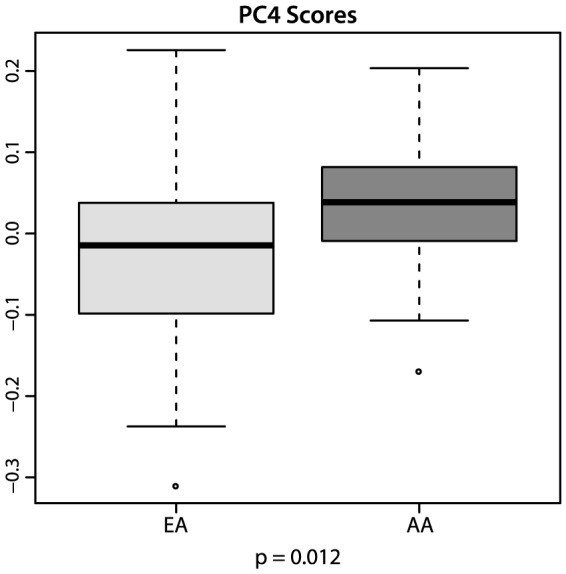
Differential projection scores on principal component 4 by ethnicity.

Samples from African American patients received significantly higher scores on PC 4 than samples from European Americans (stage-adjusted p = 0.012, Figure 1). The lower end of this component comprised many factors commonly induced by the transcriptional machinery downstream of ER such as *ANKRD30A, TFF3, GATA3* and *SFRP2* despite the absence of estrogen receptor expression in TNBC. The transcriptional regulator *FOXA1* which is a mediator of estrogen signaling in ER-positive luminal breast cancer [15] was found in the ten highest scoring genes. Gene set enrichment tests of the 250 lowest scoring transcripts (listed in Table S2) revealed signatures of stromal tissue, extracellular matrix remodeling, cell adhesion and estrogen-receptor positivity. Expression of these genes is typical of the luminal androgen receptor and the mesenchymal stem cell subtypes, both of which were associated with PC 4 (Spearman's rho = 0.68 and 0.83, respectively). Transcripts with positive contributions to this component – and therefore overexpressed in AA samples, were dominated by markers of proliferation such as *AURKB*, *CDCA5, CENPM, DDX11, MKI67* and negative ER status, indicative of the basal 1 subtype described by Lehmann and colleagues [9]. The transcriptional regulator *FOXM1* had a high projection score on this component and might be of interest for these motifs. Cued by these markers, differential activation of previously published signatures related to those in PC4, was analyzed with respect to ethnicity.

### African American patients have a transcriptomic signature consistent with BL1 subtype, BRCA deficiency and increased IGF1 pathway activation

**Figure 2:**
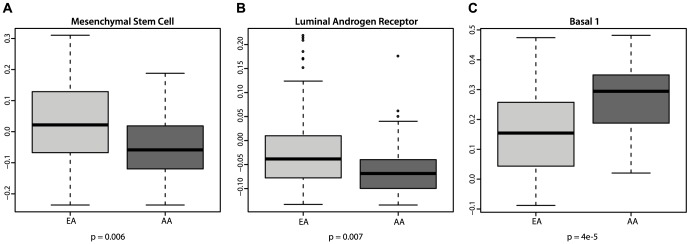
Association of ethnicity with TNBC subtypes [**9**]. Correlations of gene expression with the mesenchymal stem cell (A), luminal androgen receptor positive (B) and basal 1 (C) subtypes were compared between African American (AA) and European American (EA) patient samples.

The proliferative and differentiation-related signatures from principal component 4 were unequally activated between samples from African American and European American patients (stage-adjusted p = 0.012). We therefore examined the association of related literature-derived signatures with ethnicity. European American tumors demonstrated significantly higher scores in the luminal androgen receptor and mesenchymal stem cell signatures ([9], p = 0.007 and 0.006, respectively, Figure 2A and B). Samples with mesenchymal stem cell like gene expression also received high scores in a signature of tumors enriched with putative mammary stem cells (CD49fhi,EpCAM-, Figure S1) [16]. Such tumors share many properties with claudin-low and normal-like tumors such as low proliferation and chemoresistance. The rare luminal like androgen receptor driven subtype is characterized by expression of the androgen receptor, absence of basal cytokeratins and poor response to cytotoxic chemotherapy. *In-vitro* evidence suggests the androgen receptor pathway as suitable target [9].

**Figure 3:**
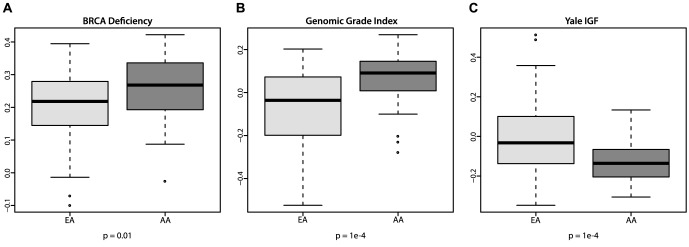
Associations of ethnicity with published gene expression signatures. Signatures of (A) *BRCA1* deficiency [37] (B) genomic grade [17] and (C) IGF1 ligand activation [18] were compared between African American (AA) and European American (EA) patient samples.

African American ethnicity was strongly associated with the basal 1 subtype (p = 4e-5), described as basal cytokeratin-expressing, highly proliferative tumors with increased cisplatin sensitivity [9] (Figure 2C). Tumors from AA patients exhibited features of genomic instability similar to *BRCA1* mutant tumors, as indicated by high activation of a *BRCA1* deficiency signature (p = 0.01Figure 3 A). Furthermore, these samples had a markedly higher genomic grade ([17], p = 1e-4, Figure 3 C) and low levels of an insulin-like growth factor 1 (IGF1) ligand signature (p = 1e-4, Figure 3 D). Low levels of this IGF1 ligand signature were associated with high grade and increased expression of IGF1 receptor in an independent cohort [18].

### Gene expression and immunohistochemistry show increased vascularization in tumors from African American patients

**Figure 4:**
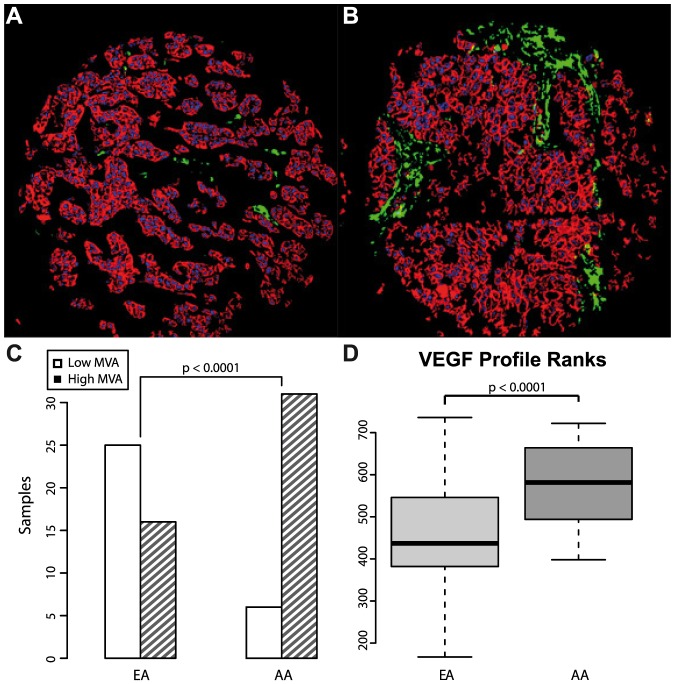
Representative histospots depicting microvessel area (MVA) and expression of angiogenesis markers. Panels (A) and (B) show cytokeratin staining for determination of the tumor area in red, DAPI-stained nuclei in blue and CD31-positive microvessel area in green. Panel A depicts a representative histospot from a European American sample, panel B shows a representative African American sample. (C) Proportion of African American and European American patients with large microvessel area (more than 0.6%, hatched bars) (D) Expression ranks of 11 VEGF-activated genes [19] in African American and European American samples.

Reports of increased lymph node and distant metastasis rates in African American patients [3], [4] led us to investigate tumor vascularization using a 13-gene VEGF profile [19] and immunohistochemistry. The VEGF profile published by Hu *et al.* defines 13 genes up-regulated in metastatic tumors of which 11 were represented in our dataset. Out of these, six showed individually increased expression in AA tumors (Bonferroni-adjusted p<0.05, Figure S3). A combined VEGF profile score created from the ranks of gene expression suggests an overall increased expression of the VEGF profile in AA samples compared to EA (p<1e-4, Figure 4D). This finding is further supported by quantification of microvessel area (MVA) by AQUA which showed significantly higher normalized vessel areas in AA samples. Using a threshold of 0.6% microvessel area, consistently found to assign samples into clinically meaningful groups [20], significantly more AA patients were assigned to the high MVA group compared to EA (stage-adjusted p<0.0001, normalized cutoff of 0.6 [20], Figure 4C). High MVA scores as quantified by AQUA are associated with higher tumor size, node positivity and hold prognostic value in unstratified breast cancer cohorts [20]. Consistent with data from unstratified breast cancer samples, microvessel area was significantly associated with lymph node status in this triple negative cohort (p<0.008, using either the threshold or numeric microvessel area), which may link to the observation that AA patients were more frequently found to be node positive than EA.

### External validation confirms differential association of ethnicity with TNBC subtypes

We used gene-mapped normalized RNA-Seq and reverse phase protein array data from The Cancer Genome Atlas (TCGA) to validate the observations made in this cohort. While the associations with specific pathways such as the IGF1 (p = 0.016) signature, the BRCA deficiency signature (p = 0.095) or the genomic grade index (p = 0.040) were statistically borderline significant and visually less pronounced, (Figure S4A–C) we could more clearly confirm the associations with TNBC subtypes published by Lehmann and colleagues despite the small number of samples (p = 0.07, 0.009 and 0.008 for mesenchymal stem cell, luminal androgen receptor and basal 1 signatures, respectively, Figure S4D–F) [9]. No protein marker of basal subtype was available, however both mRNA (not shown) and protein expression for the luminal marker GATA3 and androgen receptor (AR) were significantly higher in samples from European American patients (p = 0.002 and 0.031, respectively, Figure S5A and B).

A total of 7 out of 13 genes from the VEGF profile [19] showed a tendency towards increased expression in TCGA samples from African American patients, however no statistical significance was found for the overall profile rank (data not shown). Protein expression data for the vascular marker CD31 showed no differential expression, however since the measurement was done using lysate and is not validated as a marker of angiogenesis therefore it may be of limited value,

## Discussion

Using gene expression profiling and protein expression analysis of microvessel area to assess tumor angiogenesis, this study attempted to address molecular differences between patients of African American and European American ancestry with triple negative breast cancer. Unsupervised analysis of gene expression data in TNBC showed a prominent basal-like signature which was differentially expressed between African American and European American patients. The identified modules closely resemble the TNBC subtypes described by Lehmann *et al.* in their large pooled dataset. Absence of estrogen-signaling, BRCA1 deficiency, high mitotic activity, hypoxia and invasiveness are hallmarks of the basal 1 subtype of triple negative breast cancer. Samples classified as basal 1 exhibited increased genomic instability and responded to DNA-damaging cisplatin chemotherapy [9]. The transcriptional profile of the basal 1 subtype was associated with a low IGF1 signature score, previously shown to be a marker of high IGF1 receptor (IGF1R) expression and upregulation of the MAPK and AKT growth pathways [18]. While an increased activity of the IGF1 pathway is commonly observed in triple negative breast cancer, we observed substantial ethnicity-associated heterogeneity within TNBC samples.

A number of IGF1 receptor inhibitors are currently in preclinical and clinical trials [21]. Consistent with the predominant activation of the IGF1 pathway in TNBC, a phase II study on the anti IGF1 receptor antibody AMG 479 found no improvement of progression-free survival in patients with estrogen-receptor positive tumors [22]. In contrast, multiple preclinical studies show that triple negative cell lines and tumorgrafts are sensitive to IGF1 inhibition [23], [24]. In addition to previous data showing that the IGF1 pathway may be a sensitive target in TNBC, this study suggests that African American patients with triple negative breast cancer represent an understudied group of patients who may benefit from drugs targeting the IGF1 pathway.

African American patients received significantly lower scores for the mesenchymal stem cell like subtype than European Americans. Cell line models for mesenchymal-like TNBC are highly sensitive to inhibition of growth factor signaling by the tyrosine kinase inhibitor dasatinib and the PI3K/mTOR inhibitor NVP-BEZ235 [9]. The luminal androgen receptor (LAR) driven subtype signature was low in most samples, however 11 samples (12%) formed a distinct LAR-high cluster containing 7 EA and only one AA sample (3 unknown). Virtually the same distribution was found in the TCGA validation data (14% EA, 0% AA, combined p = 0.007, Figure S2A and B) and confirmed by differential AR protein expression. Samples of the LAR subtype express luminal rather than basal cytokeratins and are classified as luminal A or B intrinsic subtypes described by Perou *et al.* [25]. This rare subtype is consistent with the previously described “molecular apocrine” histology [26] and shows drastically different clinical behavior from most TNBC tumors, including chemoresistance and potential sensitivity to the AR antagonist bicalutamide [27], [28]. A phase II trial (NCT00468715) is currently investigating the effectiveness of bicaluamide in ER negative, AR positive breast cancer and a recent study found that AR expression is associated with a favorable prognosis subgroup of chemoresistant TNBC tumors [29]. We also found significant disparity of GATA3 expression, present at very low levels in AA samples and expressed over a wide range in EA samples from the TCGA cohort. GATA3 is a luminal marker which acts independently of estrogen receptor in TNBC. It has been associated with favorable prognosis in chemoresistant tumors [29] and suppresses the expression of basal subtype genes through interaction with BRCA1 [30]. While variants of *GATA3* are known to influence tumor biology in ER-positive breast cancer [31], their role in TNBC heterogeneity remains to be studied.

In addition to the transcriptional signatures of elevated proliferation and VEGF activation in AA tumors, automated quantification of microvessel area indicated that AA tumors are more likely to be highly vascularized. Elevated microvessel area quantified by AQUA was shown to be a marker of node positivity and reduced 20 year survival [20]. Although differences in overall survival were not observed between EA and AA patients in this cohort, this finding is consistent with higher frequencies of lymph node metastases in AA patients observed in this cohort and elsewhere [3] and may advocate the utility of antiangiogenic drugs for African American patients with triple negative breast cancer.

In conclusion, this study was able to replicate the subtypes described by Lehmann and colleagues in two independent cohorts and show significant differences of their prevalence in AA and EA patients. Moreover, the basal 1 profile activated in AA patients may be accompanied by an increased degree of tumor angiogenesis. These molecular differences in TNBC breast cancer may not only explain disparities in outcome observed between AA and EA patients but suggest that specific targeted therapy approaches should be considered in particular racial groups.

## Materials and Methods

### Study population

A cohort of 136 patients diagnosed with triple negative breast cancer at Yale New Haven and Bridgeport, CT Hospitals between 1985 and 2007 was selected from an institutional pathology database (CoPath) using ER, PR and HER2 search terms. Histology and triple negative receptor status of the tissue blocks were reviewed by the study pathologist (VB). ER, PR and HER2 testing were performed in a clinical lab by Yale pathologists as part of the diagnostic work-up. ER/PR were measured by IHC and HER2 was determined by IHC with reflex to FISH – all scoring was performed per ASCO/CAP guidelines. Patients of African American ethnicity were oversampled to allow statistically valid comparisons between ethnic groups. Following the approval of the Yale institutional review board, clinical and demographic data were extracted from the Yale and Bridgeport hospital tumor registries. In particular, ethnicity of patients was self-reported and for this study assigned to four groups (African American, European American, Hispanic and other/unknown) based on review of medical records.

### Immunohistochemistry and automated quantitative analysis (AQUA) for Microvessel Area

Tissue microarray (TMA) slides were constructed as previously described [13], [16]. Primary antibodies used were mouse monoclonal anti-CD31 (DAKO) [14] at 1∶50 with polyclonal anti-cytokeratin (DAKO) at 1∶200 in 0.3% BSA/TBS buffer for 1 h at 37°C. Alexa 546-conjugated goat anti-rabbit (1∶100) in goat anti-mouse conjugated to horseradish peroxidase (HRP)-decorated dextran-polymer (Envision; DAKO) were incubated as secondary antibodies for 1 hour. Slides were incubated for 10 min with Cy5-tyramide to the envision HRP for visualization of the target. Nuclei were stained with DAPI and anti-fade mounting media (Molecular Probes, Eugene, OR). AQUA technology was used to image specimens as described previously [13]. Briefly, slides were scanned and high resolution images were taken of each TMA spot (histospot), using the appropriate wavelengths. The cytokeratin image was used to generate a binary tumor mask distinguishing tumor from stroma. Histospots with less than 5% tumor content were excluded. DAPI images were used to assign each pixel under the tumor mask into non-overlapping non-nuclear (membrane/cytoplasmic) and nuclear locales. Finally, the target CD31 was used to create a vessel mask to determine a microvessel area (MVA) as previously described [14]. This measure has been shown to be an objective and reproducible marker of tumor vascularization with prognostic relevance in breast and other cancers [20], [32].

### Tissue processing and RNA extraction

Samples from 136 patients had formalin-fixed paraffin-embedded (FFPE) tissue blocks available for nucleotide extraction. Invasive disease was identified on H&E sections by the study pathologist (VB) and one to three 1.5 mm cores were punched from the top down in the designated tumor areas of each FFPE block. The cores were deparaffinized with xylene at 50°C for 3 minutes. RNA was extracted using the RecoverAll Total Nucleic Acid Isolation kit (Applied Biosystems) following the manufacturer's protocol.

*Whole genome mRNA expression analysis*: 110 RNA samples from 98 patients contained sufficient amounts of RNA for gene expression analysis. The isolated RNA was hybridized to Whole-Genome DASL (HumanRef8 V 3.0, Illumina) at the Yale Center for Genome Analysis.

### Data processing

Data preprocessing and statistical analysis were carried out in Bioconductor/R software. Gene expression data from three WG-DASL runs (February, March and August 2009) were combined in one expression set and processed together. Samples derived from other sites than the primary tumor were discarded and in the case of multiple samples per patient, only the best scoring sample was selected during quality control. Samples with less than 30% detectable probes and probes that were detectable in less than 10% of the samples were discarded. Due to a pronounced batch-effect per slide of 8 samples in each run, probes with clustering of consecutive undetectable samples at an average overall detection rate were discarded at a p-value threshold of 0.0001. Intensity values were log2-transformed, outliers were removed based on their distance from the sample mean and the expression set was quantile-normalized. 90 samples and 18345 probes remained after filtering. Gene expression data has been deposited under the NCBI Gene Expression Omnibus (GEO) accession GSE46851.

*Statistical analysis*: Biological information was separated from technical artifact using principal component analysis (PCA), a method commonly applied for signal separation. Principal components represent orthogonal projections of the gene expression data in descending order of their contribution to the overall variance. The technical information content of each component was assessed by correspondence to RNA quality, specifics of tissue processing and the structure of the previously described batch effect. Technical parameters found to correlate with principal component scores were time since diagnosis or an internal PCR-based estimate of RNA integrity. Correspondence of principal components with clinical parameters was assessed by Pearson correlation between the loading scores and numerical variables or by two-tailed t-test for nominal parameters (e.g. ethnicity or lymph node status). A component was judged biologically relevant if a significant number of contributing genes (, i.e., those receiving a high absolute projection score) had common functional annotations. This was quantified by the enrichment of gene sets in databases such as Gene Ontology (GO) [33] and the Molecular Signature Database at the Broad Institute (MSigDB, version 3.0) [34] using Fisher's exact test. GO enrichments were computed using the *classic* and *elim* algorithms from the topGO package for R with an elimination threshold p-value of 0.01 [35].

In order to further investigate the transcriptional modules from unsupervised analysis, we retrieved published gene expression signatures. Activation of a gene signature, computed as Pearson correlation between the signature vector and each sample's expression profile for the signature genes, requires coordinated expression of multiple probes which is especially valuable for noisy data. Analysis of signature activation was preferred over individual probes because tissues for this study had been conserved in formalin-fixed, paraffin-embedded (FFPE) blocks for up to 20 years, which led to a substantial degree of RNA degradation [36]. The significance of differential signature activation in distinct clinical groups (e.g., patients of different ethnicity) was assessed by a two-tailed t-test between the respective Pearson correlations. Unless otherwise indicated, all reported p-values were FDR-adjusted for multiple testing and controlled for stage using 1000-fold resampling of stratified groups.

### External validation in The Cancer Genome Atlas (TCGA)

TCGA provides a large number of well-annotated high-quality samples with RNA-Seq and protein expression data. Level 3 RNA-Seq (normalized and grouped counts per gene) and reverse phase protein array (RPPA) data were obtained using the Broad Institute's Firehose tool (stddata run, version 2013-04-06). We filtered the sample list down to 73 cases based on following criteria: Negative ER, PR status as annotated, negative HER2 status (staining level 0 or 1 or negative FISH at staining level 2), primary tumor sample with invasive ductal histology and African American or non-Hispanic European American ethnicity. One sample per patient was used. To control the small number of samples for stage associations, clinical stages were combined into numeric groups ranging from 1 to 4. This resulted in 47 and 10 RNA-Seq samples from European American and African American patients, respectively. Normalized counts were log-transformed to enable their use with linear Pearson correlation, and analyzed for signature activation in the same fashion as DASL microarray data. RPPA protein expression data was available for 49 samples (42 EA, 7 AA). A list of case identifiers used for validation is given in Table S3.

### Ethics Statement

The research conducted in this study was performed with tissue samples and clinical data unlinked from patient identifiers. The Yale IRB approved this study and granted waiver of informed consent.

## Supporting Information

Figure S1 **Correlation of Mammary Stem Cell** [**16**] **signature scores with the mesenchymal stem cell subtype** [**9**] **.**
(PDF)

Figure S2 **Expression of androgen receptor mRNA and activation of the luminal androgen receptor (LAR) signature** [**9**] **in the Yale TNBC cohort (A) and The Cancer Genome Atlas (TCGA).** Light gray circles indicate European American ethnicity, dark gray triangles indicate African American ethnicity and empty circles indicate unknown ethnicity. Clustering by k-means assigned 11 (A) and 8 (B) samples to a LAR high group (outlined red).
(EPS)

Figure S3 **Expression of 11 VEGF-Profile** [**19**] **genes in African American (AA) and European American (EA) patients.** Unadjusted p-values are printed below the plots and significant differences at Bonferroni-adjusted p-values below 0.05 are marked by **.
(EPS)

Figure S4 **Validation of differential expression between samples from European American (EA) and African American (AA) patients in RNA-Seq data from The Cancer Genome Atlas (TCGA).** (A) BRCA deficiency signature [37], (B) genomic grade index [17] and (C) Yale IGF1 ligand signature [18]. (D–F) TNBC subtype scores [9]: (D) mesenchymal stem cell, (E) luminal androgen receptor, (F) basal 1 subtype. P-values were determined by two-tailed t-test.
(PDF)

Figure S5 **Differential expression between European American and African American patients in protein expression data from The Cancer Genome Atlas (TCGA).** (A) Expression of GATA3 protein and (B) androgen receptor (AR) protein determined by reverse phase protein array. P-values were determined by two-tailed t-test.
(EPS)

Table S1 **Gene Sets associated with the immune signature extracted from principal components 5 and 6.** 250 highest-scoring probes from both components were subjected to enrichment analysis in the Broad Molecular Signature Database (MSigDB) [34] and Gene Ontology [33].
(XLS)

Table S2 **Genes corresponding to the 250 top-scoring probes (positive and negative) projected on principal component 4.**
(TXT)

Table S3 **Case identifiers of the samples used for validation in The Cancer Genome Atlas.**
(TXT)
