# Supplementary figures and images for: Molecular Phenotypes in Triple Negative Breast Cancer from African American Patients Suggest Targets for Therapy
Source: PLoS One. 2013 Nov 18;8(11):e71915. doi: 10.1371/journal.pone.0071915 (PMC3832509; doi:10.1371/journal.pone.0071915)

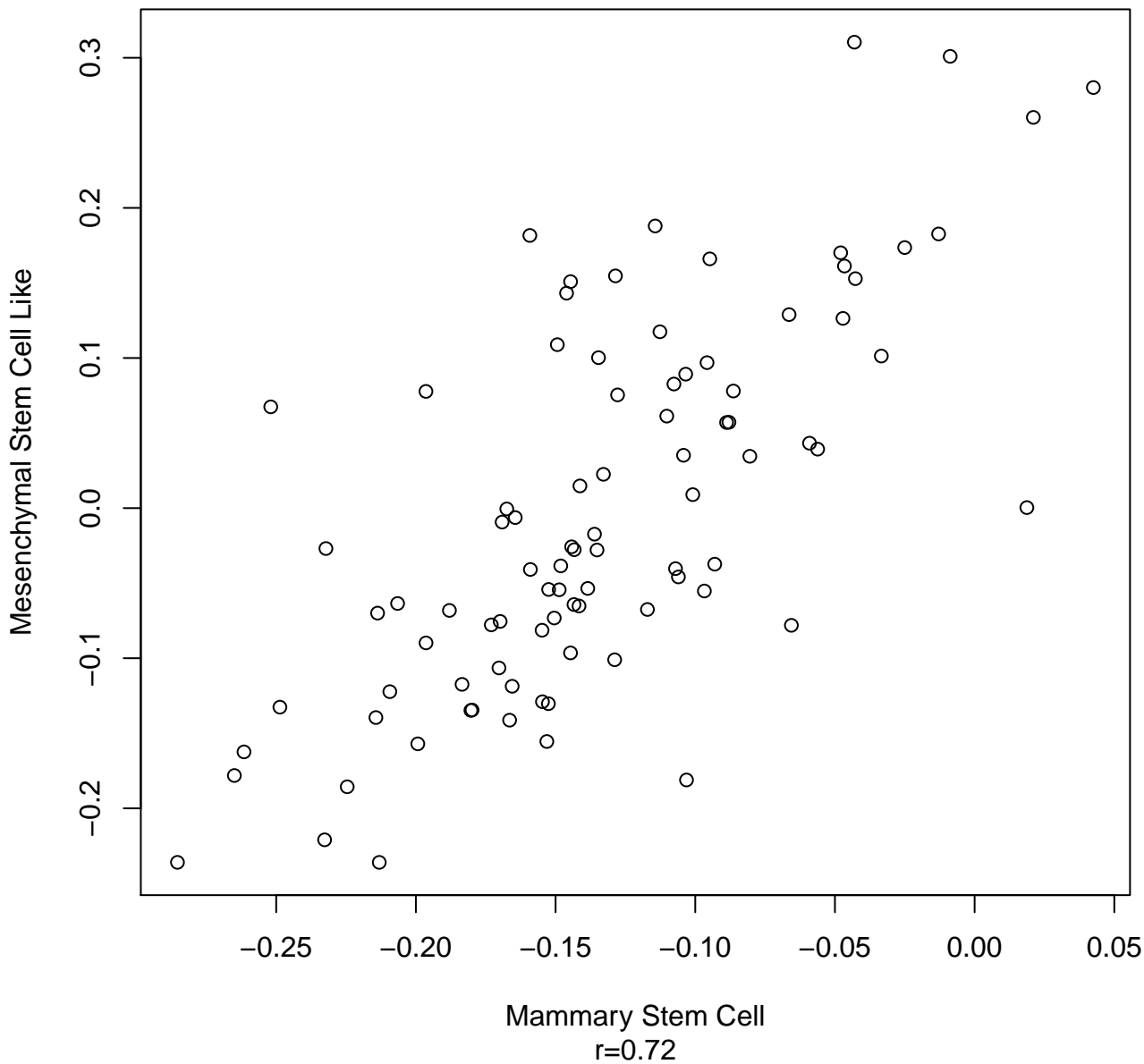

Supplement: Figure S1 — Correlation of Mammary Stem Cell [16] signature scores with the mesenchymal stem cell subtype [9] . (PDF) [file pone.0071915.s001.pdf]

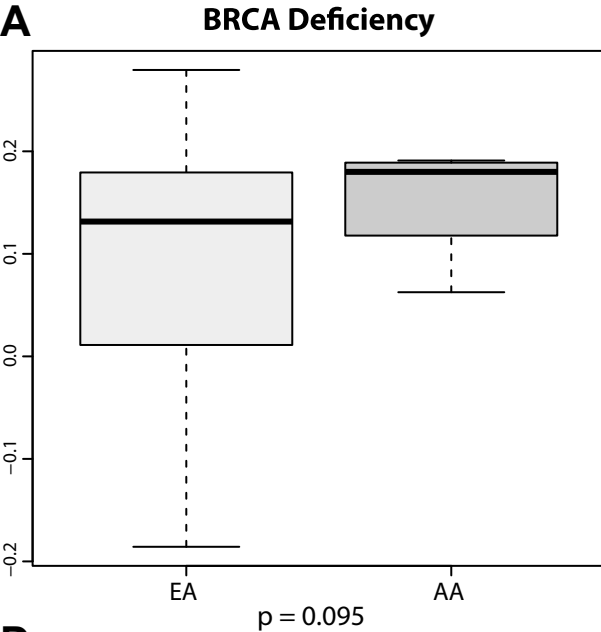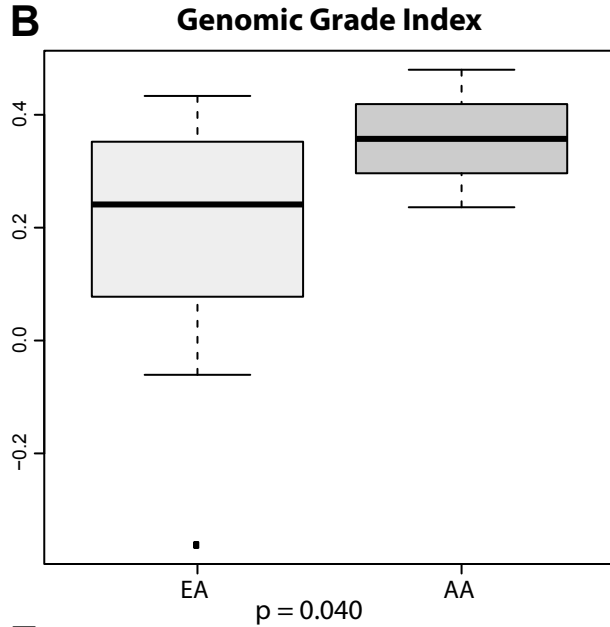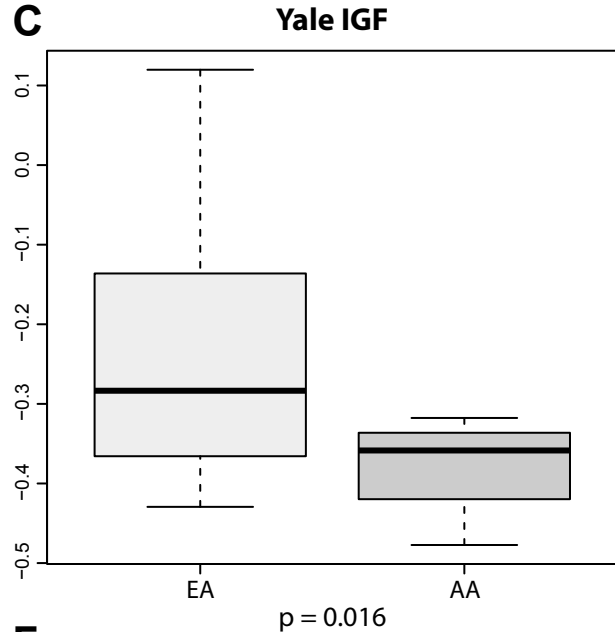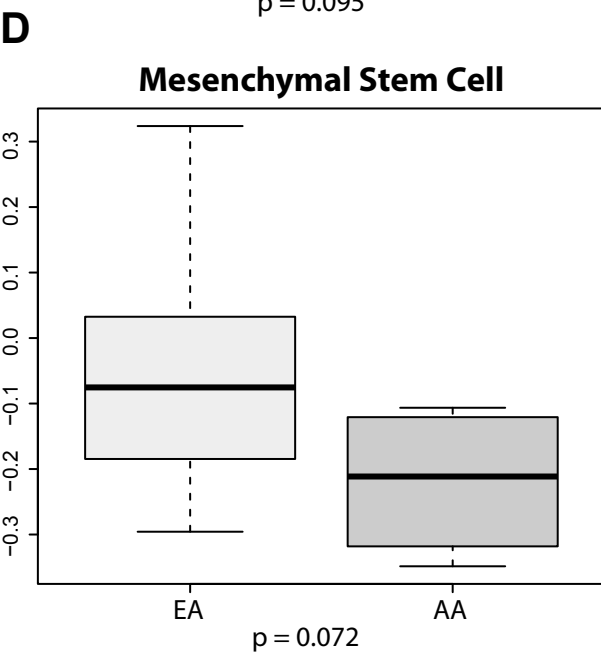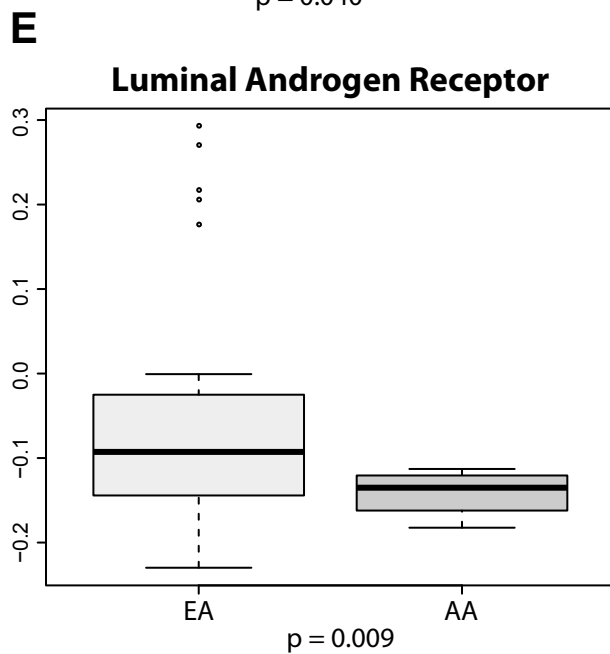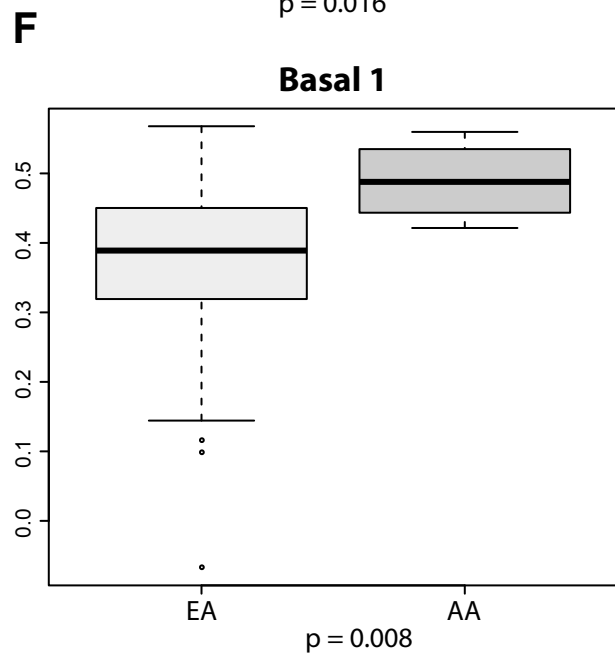

Supplement: Figure S4 — Validation of differential expression between samples from European American (EA) and African American (AA) patients in RNA-Seq data from The Cancer Genome Atlas (TCGA). (A) BRCA deficiency signature [37], (B) genomic grade index [17] and (C) Yale IGF1 ligand signature [18]. (D–F) TNBC subtype scores [9]: (D) mesenchymal stem cell, (E) luminal androgen receptor, (F) basal 1 subtype. P-values were determined by two-tailed t-test. (PDF) [file pone.0071915.s004.pdf]
